# Recent Advances in Minimally Invasive Liver Resection for Colorectal Cancer Liver Metastases—A Review

**DOI:** 10.3390/cancers15010142

**Published:** 2022-12-26

**Authors:** Winifred M. Lo, Samer T. Tohme, David A. Geller

**Affiliations:** Division of Hepatobiliary and Pancreatic Surgery, University of Pittsburgh Medical Center, Pittsburgh, PA 15213, USA

**Keywords:** minimally invasive surgery, laparoscopic liver resection, laparoscopic hepatectomy, colorectal cancer liver metastases, colon cancer, metastatic colorectal cancer, liver surgery

## Abstract

**Simple Summary:**

Minimally invasive surgery has been slowly incorporated into liver resection for metastatic colorectal cancer. Here, we review the perioperative safety and efficacy for laparoscopic and robotic approaches for patients with liver colorectal metastases. Laparoscopic liver resection (LLR) is associated with shorter hospital stays and similar post-operative complications to open techniques. This approach does not compromise oncologic outcomes or long-term overall survival. LLR allows for the earlier initiation of adjuvant chemotherapy. Studies also show that laparoscopic simultaneous resection of both colorectal and liver tumors can be safe in highly-selected patients. Early research on robotic liver resection has demonstrated a comparable safety profile to LLR and may improve the rate of R0 resection. Minimally invasive liver surgery is a safe and effective alternative for resection colorectal liver metastases in appropriately selected patients. It should be strongly considered in patients with one or two small, unilobar, and anterolateral tumors.

**Abstract:**

Minimally invasive surgical (MIS) approaches to liver resection have been increasingly adopted into use for surgery on colorectal cancer liver metastases. The purpose of this review is to evaluate the outcomes when comparing laparoscopic liver resection (LLR), robotic liver resection (RLR), and open liver resection (OLR) for colorectal cancer liver metastases (CRLM) in 39 studies (2009–2022) that include a case-matched series, propensity score analyses, and three randomized clinical trials. LLR is associated with less intraoperative blood loss and shorter hospital stay compared with OLR. LLR can be performed with comparable operative time. LLR has similar rates of perioperative complications and mortality as OLR. There were no significant differences in 5-year overall or disease-free survival between approaches. Robotic liver resection (RLR) has comparable perioperative safety to LLR and may improve rates of R0 resection in certain patients. Finally, MIS approaches to the hepatic resection of CRLM reduce the time from liver resection to initiation of adjuvant chemotherapy. Thus, MIS liver surgery should be considered in the array of options for patients with CRLM, though thoughtful patient selection and surgeon experience should be part of that decision.

## 1. Introduction

Minimally invasive surgery (MIS) has advanced the field of complex surgical oncology over the last decade. Laparoscopic liver resection (LLR) surgery has been shown to provide clinical benefits without compromising oncologic outcomes [1,2,3,4,5,6,7]. In patients with colorectal cancer liver metastases (CRLM), a case-matched series, propensity score analyses, meta-analyses, and three randomized clinical trials have compared laparoscopic and open liver resection (OLR) for perioperative safety and efficacy. Recent advances include robotic liver resection (RLR) for CRLM, repeat LLR for CRLM, simultaneous MIS colon and liver resections, MIS approaches to posterior–superior segments, and associating liver partition and portal vein ligation for the staged hepatectomy (ALPPS) approach for CRLM. Many of the recent findings have relied on single-center retrospective data, requiring careful interpretation of the data. This review examines the safety and efficacy of minimally invasive surgery (LLR and RLR) when compared with OLR for CRLM based on retrospective studies and randomized clinical trials in the last 13 years. It also reviews the limitations and remaining questions for future study.

## 2. Methods

**Figure 1:**
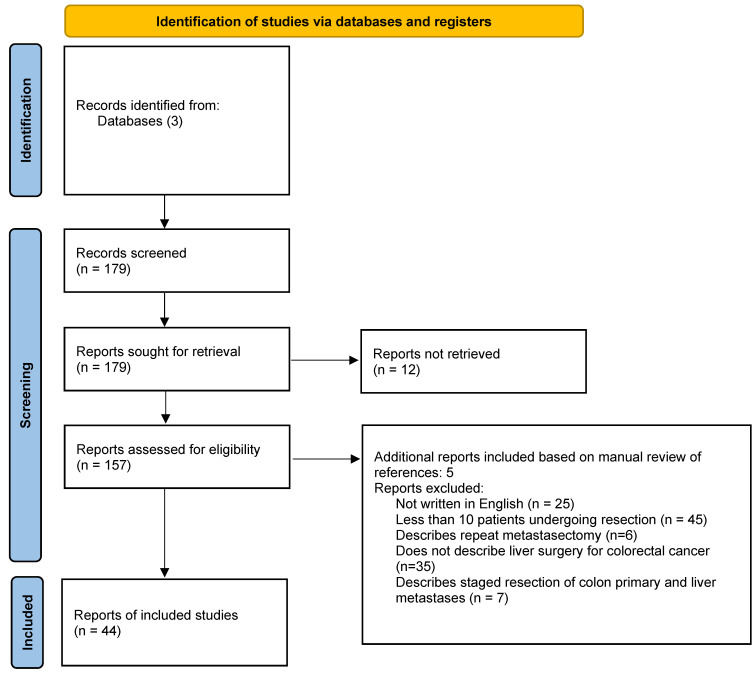
Flow diagram.

A literature review was performed using PubMed, Web of Science, and Cochrane library using the search terms “laparoscopic surgery”, “minimally invasive surgery”, “robotic surgery”, “colorectal cancer”, and “liver resection”. Papers published between January 2009 and March 2022 were evaluated for inclusion. Papers were excluded if they were not written in English, if their referenced procedures entailed only colon resection, if they reviewed liver surgery for other diagnoses, if they demonstrated outcomes not related to MIS, systematic reviews, case reports, case series regarding less than 10 patients, or if the full text could not be obtained. Conference abstracts were excluded. For papers that were review articles or meta-analyses, the reference list was manually reviewed for additional papers for inclusion. Thirty-nine papers were selected for in-depth review and inclusion (Figure 1). Data pulled from each paper included the numbers of patients, survival rates (disease-free, overall), complication rates, and mortality rates. This data was logged and reported in tables that are included for easier reference (Table 1, Table 2, Table 3, Table 4 and Table 5). If a study population performed propensity-score matching, the specific survival and perioperative safety data was extracted from that matched population.

## 3. Results

### 3.1. Retrospective Case Series Comparing LLR with OLR

LLR has been evaluated extensively for safety and efficacy in several retrospective studies. These include both single-center and multi-center analyses with characterizations of LLR alone and LLR when compared with OLR in propensity- or case-matched analyses. In total, these include 3814 patients described in 39 studies published between 2009 and 2022 (Table 1) [8,9,10,11,12,13,14,15,16,17,18,19,20,21,22,23,24,25,26,27]. This number consisted of 1833 LLR and 1981 OLR patients. It should be noted that there is significant heterogeneity in patient selection, with limited information on tumor location within the liver, proximity to major vasculature (portal pedicle, hepatic vein), or objective assessment of intraoperative technical difficulty (i.e., Iwate score). Most patients represented oligometastatic disease with prior resection of the colon primary, although a proportion of patients undergoing open resection in one study had a significantly higher rate of simultaneous colon and liver resections [22]. LLR was associated with a similar median operative time to open procedures without any significant prolongation of operating time. Laparoscopic resection was associated with lower estimated blood loss (EBL) and a shorter length of hospital stay (LOS) (laparoscopic 3–12 days versus open resection 5–14 days). Pringle maneuver application and time were not consistently reported across studies. In general, these studies concluded that LLR could be safely performed without any significant increase in operating time and could be performed with less EBL and a shorter length of hospital stay versus OLR.

When evaluating for safety, most studies reported low mortality rates (0–3.9%). Additionally, when comparing perioperative mortality between surgical approaches, there was no significant difference between LLR and OLR (Table 1). Perioperative complication rates (all grades) ranged from 8.8–41%. Eight studies noted that LLR was associated with a significantly lower rate of perioperative complications. While this was not consistently seen across all studies, it is noteworthy that there were no reports of increased perioperative complication rates with LLR.

**Table 1:** Studies evaluating laparoscopic and open liver resections (2009–2022).

Author	Year	Nation	Multi-Center	Arm	N	5-y OS (%)	*p*-Value	5-y RFS (%)	*p*-Value	Complication Rate (%)	*p*-Value	Mortality Rate (%)	*p*-Value
Castaing [2]	2009	France	yes	OLR	60	56	0.32	27	0.32	33		1.7 *	
				LLR	60	64		35		30		1.7 *	
Nguyen [3]	2009	US	yes	LLR	109	50		43		11.9		0	
Sasaki [8]	2009	Japan	no	LLR	76	64		NR		3.7		0	
Bryant [9]	2009	France	no	LLR	22	64		47		NR		0	
Kazaryan [10]	2010	Norway		LLR	110	47		NR		14.3		0.8	
Topal [11]	2012	Belgium	no	OLR	193	59.5 $	0.63	30 $	NS	29	0.02	1	0.89
				LLR	81					13		0	
Cannon [12]	2012	US	no	OLR	140	42	0.82	15	0.35	50	0.07	1 **	0.96
				LLR	35	36		22		23		0 **	
Iwahashi [13]	2014	Japan	no	OLR	21	51	NS	25	NS	9.5	0.21	0	
				LLR	21	42		14		24		0	
Montalti [14]	2014	Belgium	no	OLR	57	65	0.36	38	0.24	32	0.03	0	
				LLR	57	60		29		16		0	
Beppu [15]	2015	Japan	yes	OLR	342	68	0.30	51	NR	12	0.63	0.6 *	
				LLR	171	70		53		14		0 *	
Allard [16]	2015	France	yes	OLR	153	75	0.72	36	0.60	32.7	0.0002	3.9	0.5
				LLR	153	78		32		12.4		2	
De’Angelis [17]	2015	France	no	OLR	52	62	0.51	21	0.71	17.9	0.23	3.8	0.49
				LLR	52	76		21		17.2		0	
Hasegawa [18]	2015	Japan	no	OLR	69	57	0.53	29	0.33	24.6	0.005	1.4	1
				LLR	102	49		40		8.8		0.98	
Lin [19]	2015	China	no	OLR	36	55	0.79	38	0.86	30.5	0.599	0	NR
				LLR	36	51		27		25		0	
Schiffman [20]	2015	International	yes	OLR	368	46	NS	26	NS	33.2	0.03	0.9	0.92
				LLR	242	51		32		20.3		0.5	
Cipriani [21]	2016	UK	no	OLR	133	63	NR	16	0.24	39.8	0.002	1.5 **	0.99
				LLR	133	64		16		23.3		0.8 **	
Lewin [22]	2016	Australia	yes	OLR	138 ^	63	0.66	38	0.50	25	NR	1.4	
				LLR	146 ^	54		36		17		0	
Nomi [23]	2016	France	no	LLR	120	35.4 &		15 &		41.7	NR	0.8	
Maurette [24]	2017	Argentina	no	OLR	22	58.7 #	0.89	19 #	0.39	27	0.23	0	
				LLR	18	40 #		58 #		11		0	
Goumard [25]	2018	US	no	OLR	121	68	0.89	NR		59	0.001	0	
				LLR	43	81		NR		41		0	
Efanov [26]	2021	Russia	no	OLR	20	63	0.57	27	NR	10 ~	0.633	0	
				LLR	20	78		27		15 ~		0	NR
Nicolas [27]	2021	Argentina	no	OLR	56	77 ***	NS	20 ***	NS	16	0.3	1	NS
				LLR	26	75 ***		36 ***		2		0	

NR: not reported; NS: not significant; * 60-day mortality; ** 90-day mortality; *** 3-year results; ~ Grade 3+ complications; ^ reported as resections including multiple resections on same patient (specific breakdown not available in report); # 8-year survival; & large tumor cohort data included for reference; $: data reported for total study population only.

Oncologic outcomes are preserved with a laparoscopic approach. Five-year overall survival ranged from 36–81% in patients undergoing LLR and was not significantly different compared to patients undergoing OLR. Similarly, five-year recurrence-free or disease-free survival rates ranged from 14–53%, and were not significantly different from patients undergoing OLR (Table 1). While this is persuasive that LLR is a safe alternative to OLR, these conclusions needed to be tested in the context of a randomized control trial, leading to three studies that are described below.

### 3.2. Randomized Control Trials Comparing LLR vs. OLR

The randomized control trials comparing safety and efficacy in LLR versus OLR include ORANGE II, OSLO CoMET, and LapOpHuva (Table 2), which evaluated a total of 502 patients. Outcomes evaluated included perioperative safety, operating time, estimated blood loss or EBL, transfusion rate, hospital LOS, time to functional recovery, perioperative morbidity, perioperative mortality, resection margins, and survival.

**Table 2:** Randomized control trials comparing laparoscopic and open liver resection.

Author	Year	Nation	Study Type	Multi-Center	Arm	N	5-y OS (%)	*p*-Value	5-y RFS (%)	*p*-Value	Complications (%)	*p*-Value	Mortality (%)	*p*-Value
Wong [28]	2018	International	ORANGE II	yes	OLR	14	NR		NR		36	0.141	7.14	NR
					LLR	15	NR		NR		8		0	
Robles-Campos [29]	2019	Spain	LapOpHuva	no	OLR	97	47.4	0.82	23.9	0.23	23.7	0.025	1	NR
					LLR	96	49.3		22.7		11.5		1	
Aghayan [30,31]	2019	Netherlands	OSLO-CoMET	no	OLR	147	55	0.67	35.7	0.57	31	0.021	0.6	NR
					LLR	133	54		29.7		19		1	

NR: not reported.

#### 3.2.1. ORANGE II

This study was one of the original randomized control trials evaluating safety and efficacy in LLR versus OLR [28]. This was a multi-center, double-blind randomized control trial comparing laparoscopic versus open left lateral sectionectomy. The primary outcome was the time to functional recovery. The secondary outcomes were postoperative LOS, readmission rate, total morbidity rate, and mortality. After four years of recruitment, only 29 patients were randomized. The trial was closed due to slow accrual rate, which is the primary limitation of interpreting this trial. While the patient cohorts were not powered to assess significant differences, the descriptive data suggested similar times to functional recovery, LOS, and overall morbidities. ORANGE II is important for demonstrating the feasibility and safety of performing LLR as an alternative to OLR and served as the groundwork for multiple subsequent trials.

#### 3.2.2. LapOpHuva

This was a single-center RCT conducted in Spain [29]. Patients were randomized in a 1:1 format to either the LLR or OLR group after ensuring that they did not meet the exclusion criteria, which included a disseminated disease, large liver metastases, a tumor close to major vessels, or multiple bilobar tumors. If patients were safe and had no contraindications, they received adjuvant chemotherapy (specific regimen not reported). The primary end-point was 90-day post-operative morbidity. The secondary outcomes were the OS and disease-free survival (DFS), operating time, blood loss, transfusion rate, use of the Pringle maneuver, hospital length-of-stay, and 90-day mortality. After randomization, 193 patients were available for per-protocol analysis. For both population arms, similar numbers of patients presented with synchronous or bilobar liver metastases. Most patients had one–two tumors that were moderately sized (median diameter 3–4 cm). Similar proportions (27.2% vs. 33.3%, *p* = 0.091) received neoadjuvant therapy. There were no significant differences in the anatomic distributions of the tumors, with approximately 44% (OLR) and 41.7% (LLR) of patients presenting with tumor distributions in segments six–eight. There were similar rates of major liver resection between both arms (7.2 vs. 11.5%, *p* = 0.434), though the indices of technical difficulty could not be directly compared between both approaches. The Pringle maneuver was used more frequently in LLRs with longer occlusion times. There were no differences in operating times, EBL, or rates of blood transfusion between the OLR and LLR groups. Median hospital stay was shorter in LLR (4 vs. 6 days, *p* < 0.001). Post-op morbidity was significantly lower in LLR (11.5% vs. 23.7%, *p* = 0.025), though there were no differences in severe post-operative complications or post-operative mortality.

One advantage of the LapOpHuva trial is that the study included long-term oncologic outcomes. The median follow-up times were 36 (OLR) and 40 months (LLR). The five-year OS was 47.4% (OLR) and 49.3% (LLR, *p* = 0.82). Similarly, there were no differences in the 5-year DFS rates (23.9% vs. 22.7%, *p* = 0.23). Patients had similar rates of disease recurrence (71% vs. 67.7%, ns) between treatment groups, with no differences in distant or intrahepatic recurrences between technical approaches. At the time of data analysis, 46.4% (OLR) and 51% (LLR) of patients had died due to recurrent disease.

This trial was limited by having single-center, tertiary referral center design. By having two expert surgeons in each laparoscopic case and referencing at least 50 LLR cases prior to study initiation, the study represents a highly selected, expert surgeon population that would make these results less generalizable to the global population. Additionally, there is limited data on the number of patients successfully reaching adjuvant therapy—a common experience at many centers. Finally, a sizable proportion of patients underwent repeat resection (OLR 26, LLR 32), which confounds the estimation of OS benefit from the index resection.

#### 3.2.3. OSLO CoMET

This trial was the first to directly compare laparoscopic versus open surgical approaches for CRLM [30]. In this single-center trial, the recruited patients had CRLM that could be resected with parenchyma-sparing (less than three consecutive segments) resection without requiring concomitant ablation, vascular or biliary reconstruction, or the synchronous resection of the primary tumor. Patients were randomized two weeks prior to surgery but not informed on which approach until the day of the procedure. The operating surgeon was scheduled based on departmental availability and procedure complexity, and could change from parenchyma-sparing resection to hemi-hepatectomy or ablation at their discretion. The primary outcome was the 30-day complication rate. The secondary outcomes included conversion to laparotomy, unfavorable intraoperative incidents, operating times, blood loss, transfusion rates, and lengths of hospital stay. Patient follow-ups were performed at 1 month and 4 months after procedure.

Two hundred and eighty patients were enrolled. When reviewing background characteristics, patients in both treatment arms had similar numbers of metastases, neoadjuvant chemotherapy, rates of prior liver surgery, Iwate complexity scores, and similar rates of tumor location in posterior liver segments. Patients who underwent LLR had a lower rate of significant post-op complication (19% vs. 31%, *p* = 0.021), with one death in the open-surgery group with an uncertain cause of death at autopsy. LLR patients had lower lengths of hospital stay (53 vs. 96 h, *p* < 0.001) and less narcotic requirements (52 vs. 170 mEQ, *p* < 0.001) than OLR patients. Additionally, there were no differences in operating time, EBL, unfavorable perioperative incidents, or rates of transfusion. There was also no difference in 30-day readmission or reoperation. LLR patients had comparable oncologic outcomes, including no difference in R0 resection, R1 resection, or missed lesions. Cost-analysis was performed comparing both treatment strategies and demonstrated LLR was associated with more upfront OR costs ($5472 vs. $4762, *p* = 0.00), but did not contribute to an increased cost of initial hospital stay or additional necessary treatments at 1 or 4 months. The initial cost-savings of OLR were abrogated by costs from inpatient hospital stay, leading to no difference in short-term cost analysis for the perioperative period. Thus, LLR seemed to offer comparable immediate perioperative and cost-efficacy outcomes to OLR without compromising oncologic results. Quality of life was reported separately and evaluated physical functioning, physical role, bodily pain, overall health, emotional health, mental health, and social functioning [31]. Patients who underwent LLR had better functions in physical roles, bodily pain, and social functioning compared with patients undergoing OLR at 1 month. By four months, patients who underwent OLR still reported decreased physicality, although all other metrics were similar with LLR patients.

The long-term outcomes from the OSLO CoMET trial were released in 2021 after a minimum of 46 months follow-up [31]. In the intention-to-treat analysis, the median OSs were 80 months vs. 70 months (LLR vs. OLR, HR 0.93, CI 0.67 to 1.30, *p* = 0.67). The five-year OS rates were also similar (LLR 54% vs. OLR 55%, CI −11.3 to 12.3, *p* > 0.05). Predictors of poor OS included a poor ECOG status, lymph node involvement with the rectal primary tumor, the size of the largest liver metastasis, and the presence of extrahepatic disease at time of liver surgery. Operative approach was not a predictor of OS. The median RFSs were reported on the per-protocol analysis only and were 17 months (LLR) and 16 months (OLR). Five-year recurrence-free survival rates were 30% (LLR) versus 36% (OLR, HR 1.09, 95% CI 0.80–1.49, *p* = 0.57). The disease recurred in 62–67% of patients in both cohorts, with the most common sites of recurrence being the liver, lungs, and peritoneum. The predictors of poor RFS included lymph node involvement on the colorectal primary tumor and extrahepatic disease at diagnosis. The operative approach was not a predictor of RFS. These findings support the theory that LLR can offer a safe and oncologically sound alternative to OLR with expedited healing and improved quality of life in the immediate post-operative period. This study notably evaluated for the receipt of neoadjuvant chemotherapy, location of tumor, perceived difficulty (Iwate score) with resection, and number of lesions–features which are not consistently reported in other studies. Its limitations include the single-center and non-blinded trial design, which would make it difficult to extrapolate these results to a less-experienced center with a lower volume. As a result, additional multi-center trials evaluating whether these outcomes can be recapitulated at other centers would be very helpful to the field. For example, the ORANGE II PLUS is a multicenter trial in patients undergoing planned hemi-hepatectomy randomized to either LLR or OLR in 16 European centers. The results from this trial have not yet been published.

#### 3.2.4. Reflections on the Data—ORANGE II, OSLO CoMET, and LapOpHuva

The study investigators should be congratulated for conducting these trials which are challenging to accomplish and add critical information to the field. The most crucial element throughout these studies is the impact of patient selection. The single-institution RCTs favored patients with unilobar disease, single, smaller (<5 cm) metastases located in anterolateral segments, and who were amenable to parenchymal-sparing surgery. These patients were fortunate enough to have little disease burden, tumors away from major vessels or bile ducts, and were amenable to parenchyma-sparing surgery, which limits broader extrapolation to all patients with CRLM.

One complicating factor is the use of perioperative therapy. The use of systemic therapy, the type of regimen, the number of completed cycles, and the rates of completing all planned systemic therapy were unevenly reported between studies. Approximately 30% of LapOpHuva patients received systemic therapy, compared with 60–69% of patients in OSLO CoMET. Of note, neoadjuvant and adjuvant chemotherapy are often given to patients with resectable CRLM, although EORTC 40983 did not show any significant 5-yr OS benefit with use of 3 months neoadjuvant and 3 months adjuvant FOLFOX compared with surgery alone [32,33]. The use of neoadjuvant/adjuvant chemotherapy may be a significant confounding factor that was not accounted for throughout these surgical trials.

Finally, these studies were based at tertiary referral centers with high volumes in liver surgery, allowing for learning and expertise in laparoscopic approaches. Thus, safety and efficacy can be estimated for high-volume referral centers, but may not be reproducible when applied in the less-experienced centers. Collectively, these studies provide valuable information that can be extrapolated to similarly selected patients. LLR can be technically feasible, safe, and oncologically comparable to OLR for CRLM resection, and should be considered in patients who meet the selection criteria of the published RCTs.

### 3.3. Robotic vs. Laparoscopic Liver Resection Surgery

Robotic liver resection (RLR) has been evaluated for differences in feasibility, perioperative safety, and oncologic outcomes. Kingham and colleagues initially compared robotic liver resection to open resection in a single-institution, case-matched series [34]. Sixty-five patients underwent RLR between 2002 and 2014. Selection criteria included patients with resectable liver lesions that did not require procedures more extensive than a hemihepatectomy, have an invasion of the IVC, have an invasion of the main, right, or left portal veins, or require vascular or biliary reconstruction. Patients between both cohorts had similar rates of malignant and benign lesions, and incidence of steatosis and hepatitis were similar between both groups. Patients undergoing RLR had shorter operating times (163 min vs. 210 min, *p* = 0.017), lower blood loss (100 vs. 300 mL, *p* < 0.001), and lower rates of Pringle maneuver use (9% vs. 75%, *p* < 0.001). This was despite a similar rate of wedge or segmentectomy resections between groups. There were no differences in R1 resection (1.6% vs. 15%, *p* = 0.40), major complication rates (5% vs. 6%, *p* = 1.0), or 90-day mortality rates (3% vs. 1.6%, *p* = 1.0) [34]. In this cohort, RLR was safe and offered comparable short-term oncologic outcomes to OLR in appropriately selected patients at experienced, tertiary-care referral centers.

RLR was subsequently compared to LLR for safety and efficacy. Five different retrospective cohort studies compared the robotic versus the laparoscopic approach for CRLM resection in 1869 patients total (Table 3) [35,36,37,38,39]. One of these studies was a multi-center retrospective study at an Italian center (59 study patients) [35], and may overlap with results published from the IGoMILS registry. The reported outcomes included perioperative safety, LOS, and survival. There were no differences in the estimated blood loss (EBL), transfusion rates, or perioperative morbidities between the LLR and RLR groups. No differences were noted in five-year disease-free (38 vs. 44%, ns) or overall survival (61 vs. 60%, *p* > 0.05) rates. There were conflicting reports regarding the operating times. Rahimli and colleagues found in their series that RLR (n = 12) was associated with a significantly longer operating time (342 vs. 200 min) but a higher tendency towards R0 resection (100% vs. 77%, *p* > 0.05) compared with LLR (n = 12) [37]. In their multi-center analysis, Masetti and colleagues found no differences in operating times between the RLR and LLR groups, and RLR was associated with lower rates of R1 resection (16.9 vs. 28.8%, ns) with greater distances in surgical margins than LLR [39]. Beard and colleagues reviewed the collective experience in six high-volume, tertiary referral centers in the U.S. and Belgium [36]. Propensity matching was performed to minimize the differences between the LLR and RLR patients. The total cohort comprised 629 patients, including 115 patients who underwent RLR (2002–2017). Most procedures were parenchyma-sparing wedge resections. After matching for 115 LLR similar patients, there were no differences in reoperation rates (0.9% vs. 3.5%, ns), perioperative complications (27.8% vs. 31.3%, ns), perioperative mortality rates (1 LLR, 1 RLR from cardiac arrest), or margin statuses. After a median follow-up time of 2.8–3.1 years, there were no differences in the 5-year OSs or DFS. A separate meta-analysis evaluated seven retrospective cohort studies examining LLR vs. RLR in a cohort of 525 patients [38]. There were no differences in the perioperative complication rates, perioperative mortalities, rates of conversion to open procedure, R1 resections, blood transfusions, operating times, or lengths of hospital stay. No survival data could be extrapolated from the cohort studies.

**Table 3:** Studies comparing robotic and open liver resections.

Author	Year	Nation	Multi-Center	Arm		5-y OS (%)	*p*-Value	5-y RFS/DFS (%)	*p*-Value	Complications (%)	*p*-Value	Periop Mortality	*p*-Value
Guerra [35]	2018	Italy	yes	LLR	0								
				RLR	59	66		41.9		27		0	
Beard [36]	2019	US	yes	LLR	115	60	0.78	44	0.62	32	0.66	0.9	1
				RLR	115	61		38		36		0.9	
Rahimli [37]	2020	Germany	no	LLR	13	100 *	NS	54.9 *	NS	15.3	NS	0	NR
				RLR	12	44*		33.3 *		25		0	
Ziogas [38]	2020	International	yes	LLR	300	NR		NR		28	0.13	0.3	0.75
				RLR	225	NR		NR		18		0	
Masetti [39]	2022	Italy	Yes	LLR	953	NR		NR		20	0.906	0.3	0.792
				RLR	77	NR		NR		19.5		0	

* denotes 3-year survival data; NR: not reported; NS: not significant.

When reviewed in total, there were no differences in perioperative complications or mortalities when comparing RLR to LLR across any of the five studies. In the two studies that reported on survival data, there were no differences in five-year RFSs or OSs between the RLR and LLR approaches. In conclusion, RLR appears to be feasible, safe, and may improve margin resections without compromising survival. It is not surprising that the difference in perioperative safety and transfusion is comparable between RLR and LLR for the general patient population undergoing resection for CRLM. Patients with large tumors with close proximities to hilar structures and major vessels are less likely to be incorporated in this patient population. Additional study is warranted for evaluating tumors in difficult locations, with predicted high Iwate scores for surgical complexity, and, with time, with tumors adjacent to major vessels. Furthermore, the long-term survival results have yet to mature and be reported in major study centers.

### 3.4. Laparoscopic vs. Open Simultaneous Liver and Colon Resections for Synchronous Disease

Select patients who present with CRLM at diagnosis may be eligible for synchronous resection. Advances in the laparoscopic technique, perioperative care, and the use of systemic therapy have made it possible to attempt synchronous resection in appropriately selected patients. Eleven single-center and multi-center retrospective cohort studies evaluated whether laparoscopic simultaneous resection could be safely performed for patients with synchronous stage IV CRLM disease (Table 4) [40,41,42,43,44,45,46,47,48,49,50,51]. Of note, all of these studies were performed outside of the United States (France, Spain, Israel, UK, Italy, South Korea, and multi-national), and evaluated a total of 490 study patients undergoing laparoscopic simultaneous resections. Procedures were performed at tertiary referral centers with extensive prior experience in laparoscopic and open liver surgeries. They evaluated feasibility, perioperative safety, and survival.

**Table 4:** Studies evaluating synchronous resections of colon primary and metastatic liver tumors.

Author	Year	Nation	Study Type	Arm	N	3-y OS	*p*-Value	3-y RFS	*p*-Value	Complications (%)	*p*-Value	Periop Mortality (%)	*p*-Value
Akiyoshi [40]	2009	Japan	Single-center, retrospective	Lap	10	NR		NR		10		0	
Polignano [41]	2012	UK	Single-center, retrospective	Lap	13			90		28		0	
Hatwell [42]	2012	France	Single-center, retrospective	Lap	51	NR		NR		55		0	
Ferretti [43]	2015	International	Multi-center, retrospective	Lap	142	71.9 *		63 *		31		2.1	
Muangkaew [44]	2015	South Korea	Single-center, retrospective	Lap	55					76		0	
Tranchart [45]	2015	International	Multi-center, retrospective	Lap	89	78	0.17	64	0.13	15	1	6	0.49
				Open	89	65		52		15		0	
Chen [46]	2018	Taiwan	Single-center, retrospective	Lap	16	73	0.99	35	0.14	25	0.06	0	NR
				Open	22	48		15		36		0	
Bizzoca [47]	2019	Italy	Single-center, retrospective	Lap	17					47		0	
van der Poel [48]	2019	International	Multi-center, retrospective	Lap	61	NR		NR		15	0.237	0	1
				Open	61	NR		NR		9		2	
Perfecto [50]	2021	Spain	Single-center, retrospective	Lap	15	92.3		24		26.6		0	
Sawaied [51]	2021	Israel	Multi-center, retrospective	Lap	21	87	0.64	48	0.92	33	0.15	0	0.48
				Open	42	57		40		52		2	

* denotes 5-year survival; NR: not reported.

The laparoscopic resection of the synchronous disease was as safe as open resection. There were no differences in blood loss or transfusion rates. Two studies noted slightly longer operating times, although these was not significantly different from open procedures. About 5–8% of laparoscopic procedures required conversion to an open procedure, which was consistent across multiple multi-center trials. The perioperative complication rates ranged from 10–76%, with approximately 20% constituting major complications. There were no differences in major or minor complications between the laparoscopic and open procedures. The perioperative mortality was quite low, with only four deaths reported across all studies (one from open surgery, one due to liver hemorrhage requiring reoperation, one from multi-organ system failure, and one from acute coronary syndrome). The hospital length-of-stay was inconsistently reported, but ranged from 6–16 days for both the laparoscopic and open surgery cohorts. The rates of anastomotic leaks and hospital readmissions were inconsistently reported across all studies.

Oncologic and survival outcomes were premature for the study cohorts, as most reports had median follow-up times of 24–26 months for both the open and laparoscopic surgery groups. OS and DFS were the most common oncologic outcomes reported, but varied in reporting style (i.e., 3-year versus 5-year follow-up, disease recurrence rates, etc.), making consistent comparisons across study groups challenging. Several studies did not report patient OS or DFS at all. In the five studies that reported a three-year OS, there were no differences between the laparoscopic and open surgery groups, with rates that ranged from 48–92.3% [43,45,47,50,51]. The three-year DFS ranged from 15–64%, and was also similar between both groups. These findings suggest that laparoscopic synchronous resection is at least comparable to open resection from the perioperative safety and short-term oncologic outcome perspectives in appropriately selected patients. However, it is worth nothing that many of the technical advantages associated with laparoscopic resection, such as decreased blood loss and decreased overall complication rates, are lost when applying these surgical approaches to synchronous colon and liver resections. Additionally, not all patients are appropriate for selection for laparoscopic synchronous resection. These studies favored anterolateral liver tumors over posterior tumors.

The findings for these studies are limited by the pragmatic limitations of appropriate patient selection. This warrants careful interpretation of the literature and extrapolation. For example, there was significant heterogeneity between patient cohorts with respect to the receipt of systemic therapy. For some studies, most patients did not receive neoadjuvant therapy, and in others, less patients in the open group received adjuvant chemotherapy. Additionally, the type of systemic chemotherapy and the use of biologic agents (i.e., cetuximab, bevacizumab) were not delineated in these studies, adding potential additional heterogeneity. Most studies selected for solitary liver tumors less than 3 cm in greatest diameter, and were amenable to non-anatomic resections. Some studies specifically selected for lesions in anterolateral segments only, and specifically avoided very-low-lying rectal lesions. While these are appropriate and key factors to consider in pre-operative patient selection, it is important to understand these study limitations, especially when applying to one’s own practice. As such, there are limited evidence-based guidelines available for guidance on patient and tumor selection. One example, which nicely reviews expert consensus and evidence-based recommendations, is the Italian consensus on minimally invasive simultaneous resections [52].

### 3.5. MIS Approaches Are Associated with Shorter Times to Adjuvant Therapy

While MIS approaches to liver resection show comparable safety and efficacy to open resection, they are associated with earlier recovery and the initiation of systemic therapy. Three papers evaluated this question in retrospective cohort analyses (Table 5) [53,54,55]. Tohme and colleagues identified that patients undergoing MIS liver resection were able to start systemic therapy within 42 days after resection, as compared with 63 days in patients recovering from open resection (*p* < 0.001). These results were corroborated by Mbah and Kawai and colleagues as well. This may be because patients undergoing MIS resections experience lower rates of blood loss and perioperative complications and are more likely to have a short length of hospital stay [54,55]. Patients who experience even grade one or grade two complications may experience a delay in return to full functional capacity, thus contributing to a delay in initiating adjuvant therapy. Thus, there may be a potential advantage in using MIS approaches for liver resection to facilitate sooner recovery and the continuation of oncologic care.

**Table 5:** Studies evaluating time to adjuvant therapy after liver surgery for colorectal liver metastases.

Author	Year	Nation	N	Arm	5-y OS	*p*-Value	5-y RFS	Complications (%)	*p*-Value	Periop Mortality (%)	*p*-Value	Time to Chemo (days)	*p*-Value
Tohme [53]	2015	US	66	OLR	38	0.06	NR	38	0.19	0	1	63	0.001
			66	MIR	51		NR	26		0		42	
Mbah [54]	2017	US	44	OLR	NR		NR	36	0.03	1.6	1	39	0.0001
			76	LLR	NR		NR	14		1.1		24	
Kawai [55]	2018	France	87	OLR	NR		NR	28	0.61	0	NR	53	0.01
			30	LLR	NR		NR	33		0		45	

NR: not reported.

### 3.6. Limitations

The limitations of specific RCTs and surgical approaches were reviewed within the respective sections. However, there are some overarching limitations with our review. First, most of the published papers entailed single-center, retrospective studies from high-volume centers over extended periods of time. These inherently reflect bias from surgeons with extensive experience in laparoscopic (and robotic) colon and liver surgery and patient and tumor selection. Even within study groups, there was heterogeneity in reporting the number of lesions, the sizes of the greatest liver lesions, the unilobar versus bilobar distributions, the individual tumor locations, the anticipated technical difficulties, and whether the tumors had been treated with neoadjuvant chemotherapy. The types and numbers of cycles of systemic therapy were not universally reported, nor were the common mutational profiles (i.e., KRAS, BRAF, MSI) that are typically reviewed in patients with a systemic disease today. These reporting characteristics have significant influences on perioperative morbidity and long-term oncologic results. These data points should be considered for inclusion in prospective trials for future studies aiming to evaluate perioperative safety and oncologic outcomes.

Furthermore, several of the studies have not yet reported more-updated results for long-term survival outcomes. While OSLO-COMET and LapOpHuva reported median follow-ups of at least 40+ months, some of the retrospective cohort studies only have mature data for 3-year survival (RFS, OS). These are important to reassess as we proceed with recommending RLR and laparoscopic simultaneous resections for appropriately selected patients.

The data reported from high-volume referral centers do not imply endorsement for broad integration. Surgeons need to reflect upon their own case volume and technical competence with liver surgery, laparoscopic surgery, and robotic surgery when considering embarking on these approaches for their own patients.

Finally, there are inherent limitations to the nature of a review. The papers we identified for inclusion were selected based on search terms, identification through our selected search engines, and availability in English and in full text. Our determined exclusion criteria may have excluded reports with smaller cohorts.

## 4. Conclusions

Minimally invasive approaches to hepatobiliary surgery have made significant advances in the last 15 years. The recent literature has demonstrated that LLR and RLR can be performed with acceptable perioperative safety without adversely affecting overall survival. MIS approaches can be associated with lower blood loss, shorter hospital stays, and lower rates of perioperative complications. Furthermore, advances in laparoscopic techniques and expertise may facilitate the synchronous resection of colorectal and liver tumors in patients who present with a synchronous stage-four disease. Another benefit to LLR for CRLM is the earlier initiation of adjuvant systemic chemotherapy. The current data represents the outcomes of careful patient selection and experience in advanced laparoscopic and robotic liver surgery techniques at tertiary referral centers. As MIS liver resection continues to diffuse globally, it is hoped that additional studies will provide more data on the benefits and outcomes of laparoscopic and robotic liver resections.
